# Gallie technique versus atlantoaxial screw-rod constructs in the treatment of atlantoaxial sagittal instability: a retrospective study of 49 patients

**DOI:** 10.1186/s13018-017-0607-y

**Published:** 2017-07-11

**Authors:** Bo Yuan, Shengyuan Zhou, Xiongsheng Chen, Zhiwei Wang, Weicong Liu, Lianshun Jia

**Affiliations:** Department of Orthopedic Surgery, Shanghai Changzheng Hospital, Second Military Medical University, Shanghai, 200003 People’s Republic of China

**Keywords:** Atlantoaxial, Instability, Titanium cable, Screw-rod constructs

## Abstract

**Background:**

The objectives of this study are to investigate the clinical curative effect of Gallie technique and atlantoaxial screw-rod constructs (SRC) on atlantoaxial sagittal instability and determine the indication of Gallie technique.

**Methods:**

Data of 49 patients with atlantoaxial sagittal instability from February 2008 to May 2015 were analyzed retrospectively. The visual analog scale (VAS) score and the neck disability index (NDI) were used to evaluate the curative effect. Postoperative radiological outcomes were used to evaluate the stability of atlantoaxial joint and bone fusion. Perioperative parameters such as blood loss, operation time, radiographic exposure times, and hospital expense were also recorded and analyzed.

**Results:**

Forty-nine patients (36 men and 13 women) were included in this study. The mean age was 41.4 ± 8.9 (range from 19 to 64). All patients were followed up for 24–67 months. Among these patients, 25 of these patients underwent Gallie surgery and 24 underwent SRC surgery. The pain in the occipitocervical area of all the patients has been relieved. NDI scores and VAS scores were lower in Gallie group than in SRC group in early postoperative period. The proportion of the patients who achieved good bone fusion within 3 months after operation was 88.0% (22/25) in the Gallie group and 100% (24/24) in the SRC group. The Gallie group is lower than the SRC group in blood loss, operation time, radiographic exposure times, and hospital expense. Statistical difference was observed between the two groups.

**Conclusions:**

For patients with atlantoaxial instability who has (1) the atlantodental interval (ADI) which is bigger than 5 mm on lateral flexion-extension X-ray, or Anderson-D’Alonzo type II odontoid fracture, (2) no asymmetry between odontoid process and lateral mass on open-mouth anterior-posterior X-ray, and (3) no dislocation of lateral mass joint on the CT 3D reconstruction, Gallie technique can be chosen as a safe and effective method if atlantoaxial reduction can be achieved preoperatively. Compared with SRC, Gallie technique can relieve the pain in the occipitocervical area earlier and it can shorten operation time and reduce intraoperative bleeding, radiographic exposure times, and hospital expense effectively. However, for patients with irreducible atlantoaxial dislocation, the Gallie technique should be used with caution.

## Background

As a special part of the cervical spine in human body, atlantoaxial complex bears approximately 50% of rotary motion and 12% of flexion and extension movement of the cervical spine [1]. Without intervertebral disc between the atlas and the axis, the stability of atlantoaxial complex relies solely on the atlantoaxial joint and the transverse ligament [2, 3]. Any injury of structure above may cause atlantoaxial instability [4]. Due to its specificity in structure and position, atlantoaxial instability may cause neck pain and stiffness, activity limitation, and progressive compression of the spinal cord. Therefore, recovering normal anatomical position within a short period of time and maintaining its stability are needed for the treatment of atlantoaxial instability to prevent further spinal cord injury.

The original operation method presented by Mixter [5] and Gallie [6] was the posterior cervical wiring of lamina of C1 and C2 (Gallie technique). This technique limits the anterior displacement of atlas effectively with the principle of tension band. However, many empirical studies [7, 8] have shown that the Gallie system allowed more rotation in any direction than the atlantoaxial pedicle screw technique. Screw-rod constructs (SRC) have been the mainstream in the treatment of atlantoaxial instability. Compared with Gallie technique, the SRC technique has higher operative difficulty and incidence of vascular injury. However, not all atlantoaxial instability is accompanied by rotational instability. There are merits of Gallie technique for treatment of patients with atlantoaxial sagittal instability caused by simple transverse ligament injury or odontoid fractures.

There are few reports on the classification of atlantoaxial instability and the comparison of the wiring technique and the screw-rod technique for the treatment of atlantoaxial sagittal instability. Therefore, a comparison was conducted to investigate the clinical curative effect of the Gallie technique and the SRC technique on atlantoaxial sagittal instability to determine the indication of Gallie technique.

## Methods

### Clinical data

Data of patients with atlantoaxial sagittal instability from February 2008 to May 2015 in the Department of Spine Surgery, Shanghai Changzheng Hopital, were analyzed retrospectively.

We defined a subtype of atlantoaxial instability, which had the following radiologic features: (1) the atlantodental interval (ADI) which is bigger than 5 mm on lateral flexion-extension X-ray, or Anderson-D’Alonzo type II odontoid fracture, (2) no asymmetry between odontoid process and lateral mass on open-mouth anterior-posterior X-ray, and (3) no dislocation of lateral mass joint on the CT 3D reconstruction. The patients with these features can be diagnosed as atlantoaxial sagittal instability.

The inclusion criteria included (1) the patients who were diagnosed as atlantoaxial sagittal instability and (2) the patients who showed no symptom of spinal cord compression.

The exclusion criteria were (1) atlantoaxial rotatory dislocation; (2) arch fracture, infection, or tumor of atlas; (3) incomplete, short, absence of posterior arch of atlas, or spinal process of axis; (4) patients with severe osteoporosis; and (5) patients unable to tolerate surgery or external fixation.

### Treatment protocols

All patients underwent continuous skull traction for 3 to 14 days before operation. Atlantoaxial reduction was examined during traction with cervical lateral X-ray.

After general anesthesia with endotracheal intubation, patients were placed in the prone position on a plaster bed. Atlantoaxial reduction was confirmed with C-arm X-ray. In the Gallie group, from posterior tubercle of C1 to 15 mm on both sides and lamina and spinous process of C2 were freed by subperiosteal exposure through a standard posterior midline approach. Posterior arch of C1 was separated from dural sac. Double titanium cables passed through the front of posterior arch of C1 closely and crossed through the lower edge of spinous process of C2. Autologous iliac crest bone harvested from posterior superior iliac spine was refined to fishtail bone and grafted between decorticated lamina of C2 and posterior arch of C1. All patients were routinely immobilized with neck and chest brace for 12 weeks. In the SRC group, from posterior tubercle of C1 to isthmus on both sides and C2 and spinous process of C3 were freed by subperiosteal exposure through a standard posterior midline approach. Pedicle screws or lateral mass screws were inserted into C1, and pedicle screws or par screws were inserted into C2. After connecting the rods between the C1 and C2 screws, bone graft fusion and postoperative external fixation were taken in the same way as the Gallie group. Anterior cervical release and reduction were needed for patients who were unable to achieve reduction under traction preoperatively. Patients were placed in the prone position after general anesthesia. Scar tissue was exposed and removed between the anterior arch of C1 and dens, and around lateral mass of C1 through right anterior cervical transverse incision. Gallie or SRC surgery was undertaken after the atlantoaxial reduction through X-ray. All the operations were completed by the same treatment group.

### Clinical and radiological assessment

Clinical and radiographic data were obtained before surgery, at 1 week and 3, 6, and 12 months after surgery, and annually thereafter. Visual analog scale (VAS) and neck disability index (NDI) were used to evaluate the neck pain of the patients. Cervical spine X-ray and CT were used to evaluate the bone fusion. Delayed union was defined as fusion time longer than 3 months. If delayed union occurred, patients will need to do radiographic evaluations every month until fusion. Non-union was defined as failure to achieve fusion at 9 months or failure of fixation [9].

Perioperative parameters such as blood loss, operation time, radiographic exposure times, and hospital expense were recorded and analyzed.

### Statistical analysis

The SPSS for Window (version 16.0) was used for the analysis. For continuous variables, data were expressed as mean ± standard deviation. *t* test was used to compare continuous variables (age, follow-up duration, blood loss, operation time, total cost). Chi-square test of Fisher’s exact test was used to compare categorical variables (gender, type of instability, bone fusion rate). VAS and NDI scores at different time points within the group were compared using one-way ANONA, and pairwise comparison was conducted with LSD *t* test. VAS and NDI scores were compared using unpaired *t* test between groups. Significance was defined as *P* < 0.05.

## Result

A total of 49 patients met the inclusion criteria. Twenty-five of these patients underwent Gallie surgery and 24 underwent SRC surgery. The mean followed-up duration was 40.4 ± 13.4 months for the Gallie group and 33.3 ± 6.5 months for the SRC group. The baseline data are shown in Table 1. There was no significant difference between the two groups in terms of age, sex, type of instability, and time from injury to surgery. The patients were divided into two historical groups. In the early years of this period, most of patients underwent Gallie surgery, and in the following years, most of patients underwent SRC surgery. For this reason, the follow-up duration was significantly different between the two groups.

**Table 1:** Demographic data of the patients

Characteristic	Gallie group (*n* = 25)	SRC group (*n* = 24)	*P* value
Age (year)	40.2 ± 9.5	42.6 ± 8.2	ND
Sex (male/female)	17:8	19:5	ND
Type of instability			ND
Odontoid fracture	10	11	
Atlantoaxial dislocation	13	13	
Reoperation	2	0	
Time from injury to surgery			ND
Less than 3 weeks	6	5	
More than 3 weeks	19	19	
Follow-up duration (month)	40.4 ± 13.4	33.3 ± 6.5	<0.05

Data are expressed as mean ± standard deviation unless otherwise indicated

Intraoperative findings showed that the posterior arch of C1 of all the patients were complete. The procedures were successful, and there was no nerve damage and cerebrospinal fluid leakage. There was no infection of donor site of iliac bone after operation.

**Fig. 1:**
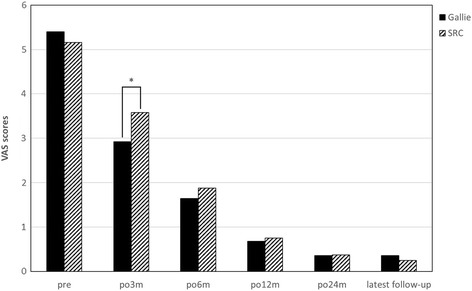
Preoperative and postoperative VAS scores. *P < 0.05

**Fig. 2:**
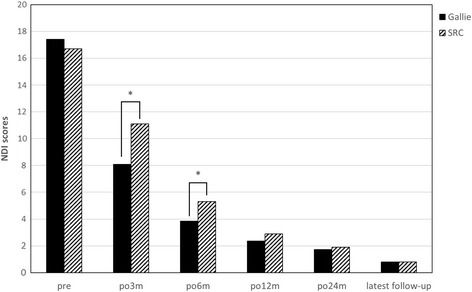
Preoperative and postoperative NDI scores. *P < 0.05

Clinical follow-up results are shown in Table 2 and Figs. 1 and 2. The pain levels in the occipitocervical areas of all the patients have been relieved 3 months after operation. At that time, the NDI scores for the Gallie group and the SRC group were reduced to 8.1 and 11.1 respectively. The VAS scores for the Gallie group and the SRC group were reduced to 2.9 and 3.6 respectively. The difference of NDI and VAS scores 3 months after operation was statistically significant between two groups.

**Table 2:** Clinical outcomes

Characteristic	Gallie group (*n* = 23)	SRC group (*n* = 20)	*P* value
NDI score			
Pre-op	17.4 ± 5.1	16.7 ± 4.9	ND
Post-op 3 months	8.1 + 1.6	11.1 + 4.0	<0.05
Post-op 6 months	3.8 + 1.7	5.1 + 1.8	<0.05
Post-op 12 months	2.4 + 1.0	2.9 + 1.3	ND
Post-op 24 months	1.7 + 1.2	1.9 + 1.1	ND
Last follow-up	0.8 ± 0.8	0.9 ± 0.9	ND
VAS score			
Preo-op	5.4 ± 1.0	5.2 ± 1.5	ND
Post-op 3 months	2.9 + 0.7	3.6 + 0.9	<0.01
Post-op 6 months	1.6 + 0.6	1.8 + 0.7	ND
Post-op 12 months	0.7 + 0.7	0.8 + 0.6	ND
Post-op 24 months	0.4 + 0.5	0.4 + 0.5	ND
Last follow-up	0.4 ± 0.5	0.3 ± 0.5	ND

Data are expressed as mean ± standard deviation unless otherwise indicated

*Pre-op* preoperative, *Post-op* postoperative, *NDI* neck disability index, *VAS* visual analog scale

Perioperative parameters are shown in Table 3. Blood loss in the Gallie group was significantly lower than that in the SRC group (*P* < 0.05). Operation time in the Gallie group was also significantly lower than that in the SRC group (*P* < 0.001). Radiographic exposure times in the Gallie group were significantly lower than that in the SRC group (*P* < 0.001). The cost of patients in the Gallie group was significantly lower than that of patients in SRC group (*P* < 0.001).

**Table 3:** Radiological evaluation of patients and perioperative parameters of patients

Characteristic	Gallie group (*n* = 23)	SRC group (*n* = 20)	*P* value
Bone fusion rate	22/25	24/24	ND
Blood loss (ml)	134.4 ± 67.4	216.3 ± 136.3	<0.05
Operation time (min)	96.6 ± 17.1	122.5 ± 17.3	<0.001
Radiation exposure times	2.2 ± 0.4	3.5 ± 0.7	<0.001
Total cost (¥^a^)	35795.3 ± 6254.0	53361.9 ± 6356.7	<0.001

Data are expressed as mean ± standard deviation unless otherwise indicated

^a^Seven Chinese Yuan equals one US dollar

**Fig. 3:**
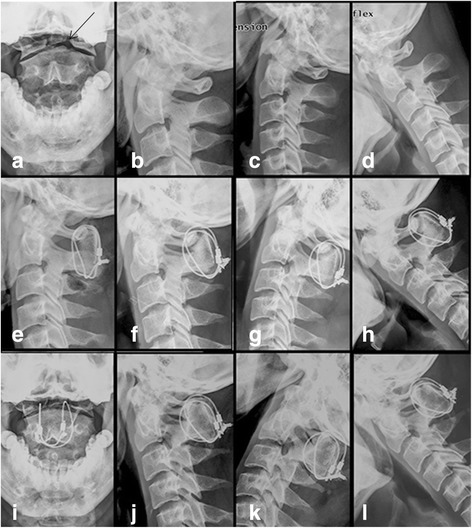
A 27-year-old man had old odontoid fracture caused by accident. **a**–**d** Preoperative X-ray showed fracture line was located at the bottom of odontoid (*arrow*), revealing an atlantoaxial instability. **e**–**l** X-ray obtained on the 1st day, the 3rd month, the 6th month, the 1st year, and the 2nd year postoperatively showed bone fusion

**Fig. 4:**
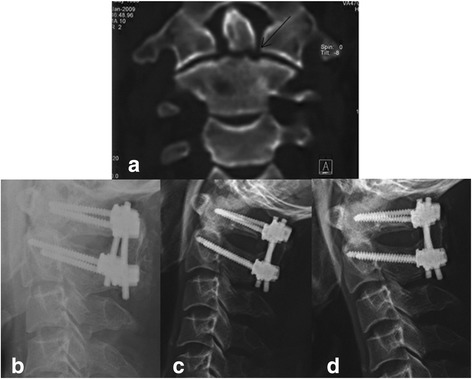
A 46-year-old man had type II odontoid fracture caused by traffic accident. **a** Preoperative computed tomography (CT) showed fracture line was located at the bottom of odontoid (*arrow*). **b** Lateral radiograph obtained on the 1st day after the surgery showed the position of C1 lateral mass–C2 pedicle screws and the autogenous bone block. **c**, **d** Dynamic lateral radiographs showed bone fusion in the 3rd month postoperatively

**Fig. 5:**
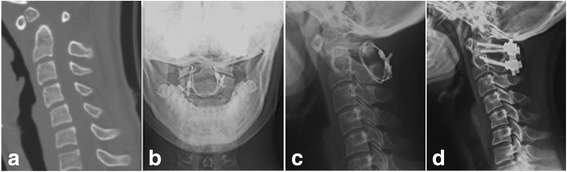
A 17-year-old woman had Os odontoideum. **a** Preoperative CT showed uncombined odontoid process. **b**, **c** Postoperative X-ray obtained at the 6th month after the Gallie fixation showed that there was no bridging bone around the iliac bone graft. **d** Lateral radiographs obtained in the 24th month after the Gallie fixation showing bone fusion

**Fig. 6:**
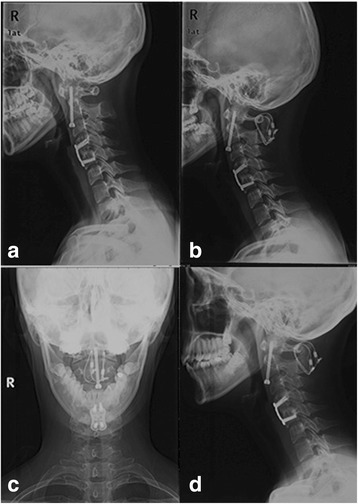
A 19-year-old woman had type II odontoid fracture caused by traffic accident. **a** Lateral radiograph showed atlantoaxial dislocation was still present. **b** Lateral radiograph obtained at 1 day after the Gallie fixation showed good reduction of atlas. **c**, **d** Open-mouth and lateral radiographs obtained at 18 months after the Gallie fixation, showing bone fusion

Radiological evaluation is shown in Table 3. Typical cases are shown in Figs. 3 and 4. Delayed union was observed in one case in the Gallie group who was asked to continue using external fixation and take radiographic evaluations on a monthly basis. He did not achieve good bone fusion until the 6th month after operation. Non-union occurred in two cases with irreducible atlantoaxial dislocation in the Gallie group who underwent anterior cervical release and reduction before the Gallie surgery. Then, they converted to the SRC surgery (Fig. 5). No deformation, displacement, or breakage of titanium cables or screws was observed in the remaining patients in the radiological evaluation. Two patients underwent the Gallie surgery due to the failure of anterior odontoid screw fixation (Fig. 6). Good internal fixation position and bone fusion were shown after the Gallie surgery.

## Discussion

The key to the surgical treatment of atlantoaxial instability is to reconstruct the stable atlantoaxial structures. Wiring fixation and bone graft fusion method like traditional Gallie and Brooks techniques [6, 10] has the advantage of being easy to perform and less traumatic to soft tissues. The Gallie technique can limit the anterior displacement of atlas effectively and enhance the sagittal stability of C1–C2. In recent years, the posterior screw and the rod system, including the transarticular screw, the pedicle screw, the lateral mass screw, the pars screw, and the laminar screw, have taken an increasingly dominant role. The atlantoaxial pedicle screw, pioneered by Goel et al. [11] and modified by Harms et al. [12], was one of the most commonly applied techniques. Moreover, the biomechanical stability of atlantoaxial pedicle screw is significantly higher than that of the wiring technique [7, 8], as well as that of the transarticular screw technique [2, 13]. In terms of the bone fusion rates, there is no distinction between the atlantoaxial pedicle screw and the transarticular screw technique [14, 15].

In the previous literatures [1, 9, 16–18], the Gallie technique was reported to have low bone fusion rate and poor clinical efficacy. According to the biomechanical studies of Papagelopoulos et al. [7] and Grob et al. [19], the Gallie technique had low resistance to rotation motion. However, the classification of atlantoaxial instability was not taken into consideration in their studies. The atlantoaxial instability can be divided into three types: anterior dislocation, posterior dislocation, and rotational instability. Simple atlantoaxial sagittal instability is defined as excessive flexion and extension movement between atlas and axis caused by odontoid fracture or transverse ligament injury etc., with or without atlantoaxial dislocation. The anatomical and biomechanical studies [7, 20–22] have shown that alar ligament and capsular ligament are primarily responsible for limiting excessive rotatory motion. When the injury of above the ligaments occurs, the range of motion in rotation of the atlantoaxial complex increases obviously. However, the atlantoaxial complex tends to increase the range of motion in the flexion and the extension when simple odontoid fracture or transverse ligament injury occurs. In addition, the atlas fracture may cause displaced lateral mass and loss of C0–C2 height, known as “buoy phenomenon” [23, 24], which may induce ligament laxity and instability of atlantoaxial complex. That was why we excluded the patients with atlantoaxial rotatory subluxation and atlas fracture.

The VAS scores and the NDI scores of 49 patients with atlantoaxial sagittal instability complex in this study were significantly lower than that in the past. The proportion of patients achieved good bone fusion within 3 months after the operation was 88.0% (22/25) in the Gallie group and 100% (24/24) in the SRC group. Good bone fusion rate in the two groups showed no significant statistical difference. Delayed union was observed in one case in the Gallie group, who achieved good bone fusion in the 6th month after the operation. This coincides closely with study of Lin et al. [9]. Non-union occurred in two cases with irreducible atlantoaxial dislocation in the Gallie group. Old dislocation resulted in a lot of scar tissues and contracture of ligament, even bony fusion. Even though reduction can be achieved after the anterior cervical release surgery, the cables tended to deform due to huge long-term sagittal stress [25]. Therefore, the Gallie technique should be used with caution for patients with irreducible atlantoaxial dislocation. The Gallie technique is able to establish stable atlantoaxial complex for patients with atlantoaxial sagittal instability caused by simple odontoid fracture or transverse ligament injury. On the contrary, in terms of blood loss, operation time, radiographic exposure times, and hospital expense, the Gallie technique is significantly better than the SRC technique. This is associated with small exposure, less damage to attachment of atlantoaxial muscle, and low cost of titanium cable. Compared with the SRC technique, the Gallie technique can relieve the pain in the occipitocervical area earlier.

In addition, the complication rates of the Gallie technique is also lower than that of the SRC technique. The insertion of C1 lateral mass screw may cause irritation of nerve root of C2. Lee et al. [26] investigated the feasibility of C1 pedicle screw fixation in patients whose atlas vertebral artery groove height is less than 4 mm. Huang et al. [27] attempted to choose a higher entry point to prevent occipital neuralgia.

Four C1 lateral mass screws were inserted in the patients in the SRC group. Although no irritation of nerve root of C2 occurred, the average amount of bleeding during operation was 216.3 ± 136.3 ml, of which the standard deviation was much larger than that of the Gallie group. Injury of the venous plexus was seen in two patients during operation, and the bleeding volume was more than 500 ml. The SRC technique may cause uncontrollable bleeding of the venous plexus.

Besides, though biomechanical strength of the Gallie technique was not ideal compared with the SRC technique [7, 13], aided by external fixation brace and excellent health guidance, no breakage of titanium cables was observed in our study. Both the Gallie technique and the SRC technique could establish stable atlantoaxial complex for patients with atlantoaxial sagittal instability. The Gallie technique distinguishes from the SRC because it can shorten the operation time, reduce intraoperative bleeding, and accelerate recovery period.

## Conclusion

Gallie technique can be chosen as a safe and effective method if atlantoaxial reduction can be achieved preoperatively for patients with atlantoaxial instability who have (1) the atlantodental interval (ADI) which is bigger than 5 mm on lateral flexion-extension X-ray, or Anderson-D’Alonzo type II odontoid fracture, (2) no asymmetry between odontoid process and lateral mass on open-mouth anterior-posterior X-ray, and (3) no dislocation of lateral mass joint on the CT 3D reconstruction. Compared with the SRC technique, the Gallie technique can relieve the pain in occipitocervical area in a shorter period of time. It can also reduce intraoperative bleeding, radiographic exposure times, and hospital expense effectively. However, for patients with irreducible atlantoaxial dislocation, the Gallie technique should be used with caution. Although aided by external fixation brace after Gallie surgery, these patients may occur non-fusion.

## Abbreviations

ADI: Atlantodental interval
NDI: Neck disability index
SRC: Screw-rod constructs
VAS: Visual analog scale
